# Wild jackdaws learn to tolerate juveniles to exploit new foraging opportunities

**DOI:** 10.1098/rsbl.2025.0179

**Published:** 2025-08-20

**Authors:** Josh J. Arbon, Noa Truskanov, Emily Stott, Guillam E. McIvor, Alex Thornton

**Affiliations:** ^1^ School of Biological Sciences, University of Bristol, Bristol, UK; ^2^ Centre for Ecology and Conservation, University of Exeter, Penryn, Cornwall, UK; ^3^ School of Zoology, Tel Aviv University, Tel Aviv, Israel; ^4^ Department of Behaviour and Cognition, University of Vienna, Vienna, Austria

**Keywords:** learning, generalization, tolerance, information use, social, flexibility, social information, social structure, aggression

## Abstract

Social tolerance can enhance access to resources and is thought to be crucial in facilitating the evolution of cooperation, social cognition and culture, but it is unknown whether animals can optimize their social tolerance through learning. We presented wild jackdaws (*Corvus monedula*) with a novel social information problem using automated feeders. Juveniles could always feed (simulating a situation where juveniles were sources of information about a new resource) but adults could only access food if they inhibited their tendency to displace juveniles and instead showed tolerance by occupying an adjacent feeder perch. Accordingly, adults learned to tolerate juveniles, with some evidence they generalized across juveniles as a cohort. The ability to learn to tolerate sources of valuable information, and generalize across cohorts of informed individuals, may facilitate adaptive responses in the face of environmental change and help to explain the success of jackdaws in human-dominated environments.

## 1. Introduction

Social tolerance, broadly defined as close proximity to conspecifics with limited aggression [1], is thought to be crucial to the evolution of prosociality [2] and social learning [3] in non-human animals, and underpins distinctive forms of cognition, cooperation and culture in humans [4]. Tolerating others can provide opportunities to learn important information such as how to obtain food [3], and may even help buffer the costs of ecological disasters [5], but social tolerance can also entail costs such as foraging competition [6]. General heuristic-based strategies can aid in responding adaptively to environmental change [5], such as preferentially tolerating kin when resource availability is low [7]. However, if potential partners vary in the benefits they provide, then learning to tolerate specific individuals, or classes of individuals, could be vital in facilitating access to information and resources [8,9]. Despite these important implications, studies have yet to explicitly test whether animals in natural social networks can learn to tolerate others based on their value, such as the information they provide.

Biases in social information use and social learning are well described across taxa [10]. Social information use entails using the presence or behaviour of others to reduce uncertainty; for instance, about where to find food or breed [11,12]. This may or may not lead to social learning (where information is later recalled in the absence of the original social cue, e.g. great tits learning a particular foraging technique from trained demonstrators [13,14]). Social information use and social learning are thus not interchangeable terms [15], but in both cases, animals are expected to be selective in who they pay attention to and when [16,17]. Such strategies are not necessarily cognitively demanding: animals need not be aware of strategies they implement, nor understand how they work for them to be adaptive [18]. One common strategy across many species is to pay particular attention to the presence or actions of older, more experienced individuals [19,20] that are more likely to provide reliable information. However, sources of useful information may change, and recent work indicates that animals can adjust their social associations based on information about specific partners [21–23]. For example, jackdaws *Corvus monedula*, flexibly alter their social associations based on past social foraging payoffs [21], and in some primates individuals receive more affiliation if they are able to access food from novel foraging tasks [22,23]. However, whether such associations can be generalized to classes of individuals that share characteristics remains unknown. In humans, for instance, younger generations commonly learn from their elders, but the digital revolution has flipped this pattern with older people seeking vital skills from younger ‘digital natives’ [24]. Similar patterns could occur in animals. Juveniles are often easily displaced by adults and generally not used as preferential information sources [25–27], but juveniles are often more exploratory and innovative [27–31]. Adults may therefore benefit from learning to tolerate juveniles to better access information they provide, especially in changeable or heterogeneous environments where reliable information sources can change quickly [32–34]. We examined this possibility using an automated field experiment on wild jackdaws.

Jackdaws are social corvids that forage in fission–fusion social groups [35] and vary in their social tolerance of different partners, generally showing particularly high tolerance to close associates and kin [36]. Under normal conditions, foraging adults do not preferentially associate with juveniles other than their own offspring (electronic supplementary material, Age-class Assortment), and often displace juveniles from foraging resources [26,36,37]. Jackdaws are proficient social learners about novel foods [38] and anthropogenic dangers [39], and have been shown to generalize learned information in foraging contexts [26]. However, juveniles are not often used as sources of information [26]. Therefore, whether jackdaws can learn to adjust their tolerance or generalize this learning across individuals, remains unknown.

To answer these outstanding questions, we used automated feeders to simulate a scenario in which juveniles are sources of information about the availability of food. While juveniles could always access feeders, adults could only feed when co-occupying the adjacent connected feeder. If an adult arrived alone or co-occupied the feeders with another adult, feeders remained locked, preventing access to food. Adults could sometimes obtain a smaller reward (max. one beakful) by displacing juveniles and accessing the food during the approximately 1 s it took for the apparatus to close, but to obtain maximum benefits, they had to refrain from displacing juveniles and instead tolerate their presence on the adjacent feeder (mean co-occupation duration ± s.e. = 12.3 ± 1.5 s).

To test whether adults learned to tolerate juveniles to exploit this novel scenario, we modelled changes in both co-occupation of feeders with juveniles and displacement of juveniles relative to adult experience, taken together as tolerance. We examined both how experience with a specific individual and experience across all juveniles, affected tolerance, to investigate learning to tolerate specific individuals versus generalizing learned tolerance to juveniles as a whole. We also tested if changes in behaviour carried over into a foraging scenario where adults gained no rewards for tolerating juveniles to investigate the potential for general increases in tolerance outside of the direct experimental context.

## 2. Methods

### (a) Study system

Experimental sessions from approximately 05.30 to 12.00 between 19 July and 13 August 2021, following the breeding season, at our long-term study population in Cornwall, UK (50°11′23″ N, 5°10′54″ W). The Cornish Jackdaw Project monitors a core nestbox population of approximately 80 boxes as part of the wider jackdaw population. Individuals are fitted with a unique set of coloured leg rings, one of which contains a radio frequency identification (RFID) tag (IB Technologies, Leicester, UK), enabling remote detection via antennae at feeders. Approximately 150 chicks from nestboxes are ringed each year before fledging, and non-nestbox juveniles are ringed as part of routine trapping [40].

### (b) Experimental apparatus

**Figure 1.**
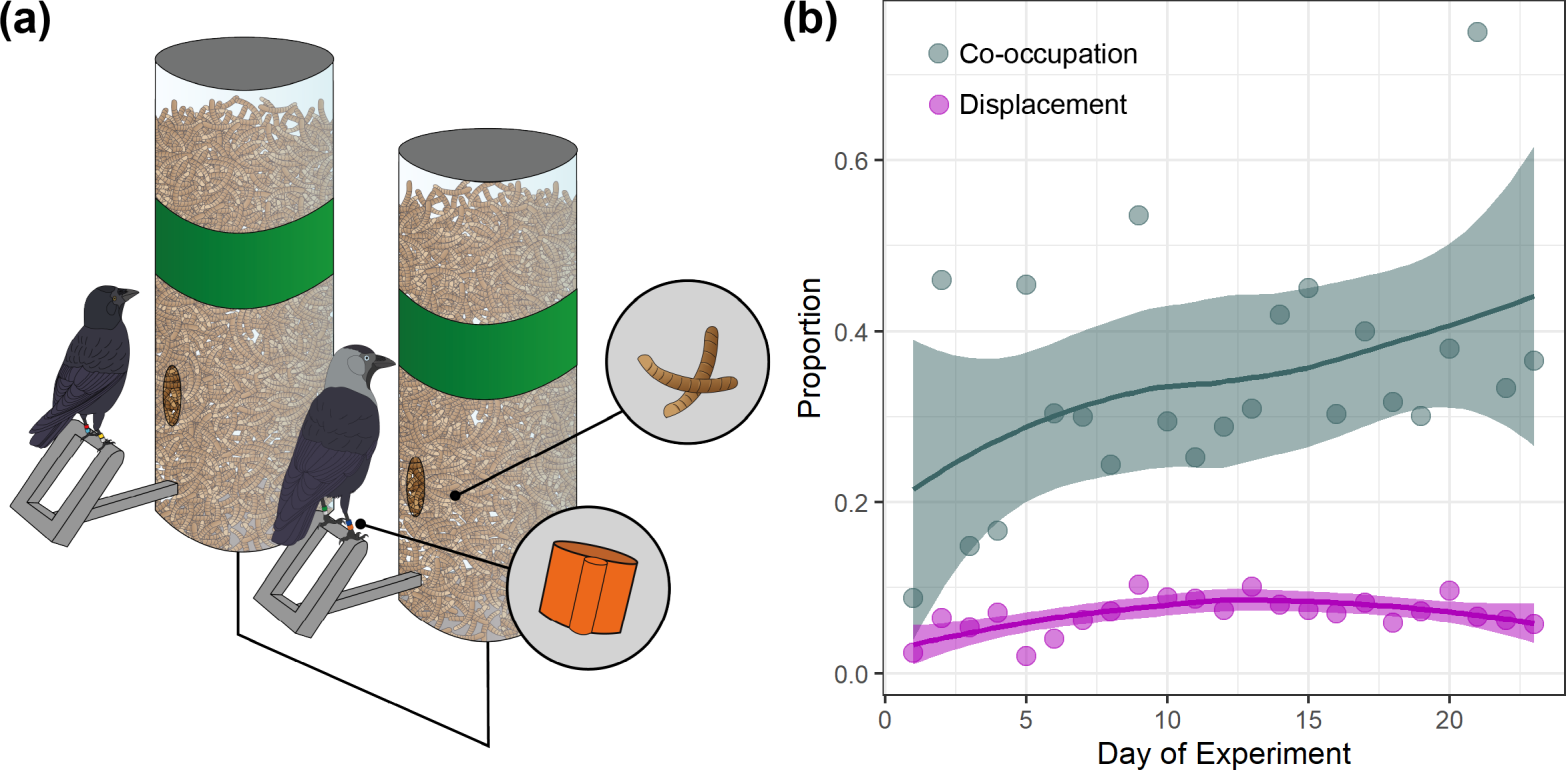
(a) *Experimental array*, showing an adult (right) in a successful co-occupation with a juvenile. RFID tags (lower circle), determine access to mealworms (upper circle) by motor-controlled doors. (b) The daily proportion of visits where adults successfully co-occupied *experimental arrays* with, or displaced, a juvenile.

Feeders automatically recorded the identity of any visiting RFID-tagged bird. Access to food was restricted by a motor-controlled door connected by a logger unit to an antenna in the perch (Naturecounters, Maidstone, UK; figure 1a; electronic supplementary material, Experimental Setup). Co-occupation events were determined from the temporal overlap between tag visits at adjacent, connected feeders (figure 1a), with the initiator and joiner determined by the time of arrival. Displacements were characterized as events where a bird was detected arriving at a feeder within 2 s of a different bird departing from the same feeder (validated through video; electronic supplementary material, Displacement Validation).

The core apparatus of the experiment were *experimental arrays*, which comprised two feeders (50 cm apart and made visually distinctive by a green band; figure 1a) containing high-quality food (dried mealworms). These feeders were connected such that the combination of tags detected across both feeders could be assessed in real time by a microcomputer, which implemented the experimental conditions. These feeders were flanked by two *flanking* feeders (electronic supplementary material, Experimental Setup) with no green band, containing low-quality food (grain). These were accessible to all individuals and served only to attract birds to the arrays. Data from these *flanking* feeders were not part of our *a priori* predictions and were not analysed for this study; all further discussion of *experimental arrays* refers only to the two central feeders that implemented the experimental conditions. Two *experimental arrays* were placed approximately 200 m apart at a mixed arable/livestock farm. For each *experimental array*, we also placed a corresponding pair of feeders with low-quality food and open access to all tagged birds (hereafter *passive arrays*) in the same arrangement: 50 cm apart, 50 m away from and out of direct lines of sight of *experimental arrays*. These *passive arrays* had no green band (making them visually distinct from *experimental arrays*) and were used to detect co-occupations and displacements outside of the direct context of the experiment and throughout the day.

### (c) Treatments

All RFID-tagged juveniles (under 1 year old) had unrestricted access to the high-quality feeders at *experimental arrays*. Adults (over 1 year old) in the Treatment group (*n* = 61 individuals detected at feeders) could access rewards from *experimental arrays*, but only if a juvenile (*n* = 22 detected) was simultaneously co-occupying the adjacent feeder in the *experimental array*. Control adults (*n* = 20 detected) could not access food from *experimental arrays* under any circumstances. All three groups had unrestricted access to low-quality food, both from *flanking* feeders within *experimental arrays* as well as the separate *passive arrays*. The control adults were intended to provide baseline data on adult–juvenile interactions, but visited *experimental arrays* at rates too low to allow meaningful analyses (electronic supplementary material, Feeder Visitation). However, the use of a control group is not necessary for our key aim of detecting changes in within-individual behaviour over time (§2(e)).

### (d) Ethical note

Research was conducted under ethical permission from the Penryn Campus Ethics Committee (eCORN000406), following Association for the Study of Animal Behavior ethical guidelines [41]. Birds were ringed under BTO permits C6449/C5752/C6923/C6079.

### (e) Statistical methods

We analysed data using relational event models (REMs), a form of time-to-event model that accounts for the structure of the social network, to compare changes in the rates of co-occupation and displacement events based on individual experience (following Kings *et al*. [21]). REMs account for the order of events in a network, preventing data aggregation [42–44], which enables us to ask questions about individual behaviour relative to past experiences, while accounting for the potential social partners available. REMs test hypotheses by comparing observed events against a set of permuted ‘non-events’ that could hypothetically happen between individuals in the network; 10 000 permutations were conducted ([45], electronic supplementary material, Relational Event Modeling, for details). All results are reported as incidence rate ratios (median and 95% confidence intervals) such that an IRR of 1.05 for a predictor variable means that the event type in question is 5% more likely for every unit increase in that predictor.

We used this approach to separately model whether adults changed their propensity to (a) join co-occupations at feeders with juveniles (electronic supplementary materials, tables S1, S3 and S5), and (b) displace juveniles (electronic supplementary materials, tables S2, S4 and S6), based on their previous information gathered at *experimental arrays*. When investigating the likelihood of an adult joining a juvenile in a co-occupation, we considered the number of times an adult had previously co-occupied with a *specific individual* (*IRR individual*), as well as the number of times an adult had co-occupied with *any juvenile* (*IRR all juvs*) to investigate generalization of learning. The likelihood of displacement was modelled as for co-occupation, with the addition of a factor for the number of times an adult had previously displaced a juvenile (*IRR displacement*) to investigate whether learning from displacements reinforced this behaviour. We ran identical models for co-occupation and displacement, relative to experience at *experimental arrays*, on data from *passive arrays* to test for carry-over effects to this context.

## 3. Results

During the experiment, we detected 2431 co-occupation events at *experimental arrays*, where both high-quality feeders were occupied simultaneously. At *experimental arrays,* all instances where an adult joined another already-present bird in a co-occupation were modeled (1665 joins of adults, 174 joins of juveniles), as well as 1997 displacements by adults (887 of adults, 1110 of juveniles). In addition, we modelled 1100 co-occupation events and 1232 displacements at *passive arrays*.

When investigating adult behaviour at *experimental arrays*, results were qualitatively identical (electronic supplementary materials, tables S1 and S2) when prior adult experience was modelled as (a) the number of prior co-occupations where that adult had joined an already-present juvenile (*n* = 174 co-occupations, 29 adults, mean/median = 6/3 per adult), or (b) as total adult–juvenile co-occupations, including when juveniles joined already-present adults (*n* = 592 co-occupations, 35 adults, mean/median = 16.9/9 per adult). We present the results from the former unless otherwise specified. Overall, co-occupations between adults and juveniles comprised 200 unique dyads, and of the 174 events where an adult joined a juvenile, only three were instances of a parent joining its own offspring.

**Figure 2.**
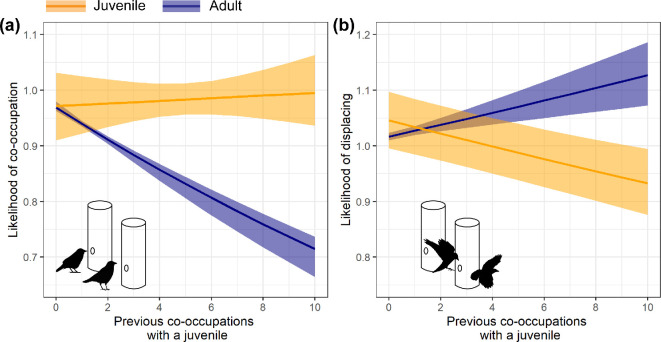
The difference in likelihood of an adult (a) co-occupation with, and (b) displacing a juvenile (orange) as opposed to another adult (blue) as adults acquired experience of joining co-occupations with juveniles. Lines and shaded areas represent median and 95% REM estimates.

Overall, the proportion of adult co-occupation events with juveniles (i.e. successes) roughly doubled across the course of the experiment (figure 1b). At the individual level, the likelihood an adult joined an already-present juvenile at the feeders (as opposed to joining another adult) increased by 3.4% for each previous successful co-occupation the adult had participated in with any juvenile (*IRR all juvs* = 1.034, *CI =* 1.021−1.049; figure 2a; electronic supplementary material, table S1a). Displacements of juveniles also became slightly more common in the first half of the experiment; the likelihood an adult displaced a juvenile increased by 0.4% with each displacement of a juvenile an adult conducted (*IRR displacement =* 1.004, *CI* = 1.002−1.006; figure 1b; electronic supplementary material, table S2a). However, the likelihood that an adult displaced a juvenile (relative to another adult) declined by 2.2% per successful co-occupation with any juvenile (*IRR all juvs =* 0.978, *CI* = 0.970−0.987; figure 2b; electronic supplementary material, table S2a).

In contrast, there is no clear evidence that experience with specific juveniles (as opposed to any juvenile), affected the relative likelihood of co-occupation with (*IRR individual =* 1.002, *CI =* 0.986−1.020; electronic supplementary material, table S1a) or displacing (*IRR individual =* 0.992, *CI* = 0.983−1.001; electronic supplementary material, table S2a) a juvenile relative to another adult.

One adult was responsible for a large number of successful joining events (37/174). If we exclude this adult from analyses, the effect of generalization across juveniles becomes less clear for patterns of co-occupation; the estimate for the effect of experience with *all juveniles* is still positive, but the CI crosses 1 (i.e. no effect: *IRR all juvs* = 1.012, *CI* = 0.987−1.044; electronic supplementary material, table S3), while the *specific individual* effect becomes more clear (*IRR individual* = 1.039, *CI* = 1.007−1.079). However, the effects for displacement are qualitatively the same without the influential adult, with an increase in magnitude for the effect size of experience with all juveniles (*IRR individual* = 1.000, *CI* = 0.988−1.012*, IRR all juvs* = 0.952, *CI* = 0.938−0.968; electronic supplementary material, table S4).

We also found some evidence that tolerance learned at the *experimental arrays* carried over to the non-experimental context. We did not see clear changes in co-occupation at *passive arrays* (electronic supplementary material, table S3); however, adults were less likely to displace juveniles if their successes with all juveniles at the *experimental arrays* were higher (though only in models where the predictor for prior experience included all adult–juvenile co-occupations; electronic supplementary material, table S4).

## 4. Discussion

Taken together, our results show that adult jackdaws can learn to tolerate juveniles when it is beneficial. In the experiment, juveniles were a source of social information: their presence at *experimental arrays* indicated the availability of high-quality food. As they gained experience of the experimental payoff structure, adults increased co-occupation with and reduced displacement of juveniles relative to other adults, indicating they learned to tolerate juveniles based on their value in reducing uncertainty about food availability. Although there were small rewards available for displacing juveniles that provided minor reinforcement for aggressive behaviour, these were greatly outweighed by the benefits of tolerant co-occupation. Moreover, our results suggest the potential for generalization across individuals to facilitate rapid, beneficial changes in tolerant behaviour, which could lead to the implementation of a new social information-use strategy, namely, ‘tolerate juveniles’.

The potential benefits of such learned tolerance are abundant. Tolerating conspecifics has been shown to facilitate cooperation in ravens *Corvus corax* [2], while tolerance is implicated in the social learning of foraging skills such as tool use in other corvid species [46,47], even in species not known to use tools in the wild [48]. Our results also mirror the increases in affiliation seen in ring-tailed lemurs *Lemur catta* and Guinea baboons *Papio papio* towards individuals that possess novel, beneficial information [22,23]. While information can be gathered from others without tolerance (e.g. broad-scale patch discovery observed from a distance), social tolerance can increase the chances of co-occurring with valuable individuals and observing their actions in close proximity. Thus, the benefits of associating with informed individuals can be achieved without any need to ‘understand’ (in a psychological sense) the knowledge they possess [18,49]. This may be especially valuable in species with fluid social groups, such as jackdaws and other animals living in fission–fusion societies, where individuals can change those they associate with if they exhibit less aggression or provide better payoffs [21,35].

While cautious about the strength of the effect, we suggest that generalizing from experience of interacting with juveniles as a cohort could be an important driver of behavioural change. By learning that the presence of juveniles in general (rather than any specific juvenile in particular) provides information about the availability of food, adults could rapidly exploit a valuable new resource. Information-use strategies are known to be influenced by developmental stress [50], but our findings highlight that learning may enable more rapid adjustment of strategies to novel conditions. We also found some evidence that changes in behaviour at the *experimental array* were reflected at passive feeders, indicating that learning had impacts beyond the context of the rewarded experiment. Whether the changes we detect in the context of foraging behaviour carry-over into other contexts (i.e. whether information use is altered more generally) is a major outstanding question. Similarly, whether such change in information use may influence social learning is unknown, as our experiment did not test whether adults learned new skills (e.g. novel foraging techniques). In tufted capuchins *Sapajus apella,* observational evidence suggests that nut-cracking behaviour was initially more common among juveniles, but the behaviour spread via a ‘learn from older’ strategy when those proficient juveniles became adults [27]. If individuals had instead switched to a ‘learn from juveniles’ strategy, this might have enabled more rapid acquisition and spread of this valuable behaviour; experiments testing the potential for such flexibility will be important in understanding how animals respond to environmental change [33,34].

Our findings are consistent with suggestions that rather than evolving as fixed, species-typical traits [10], social information-use and learning strategies use domain-general associative learning processes to modify associations and inhibit aggressive behaviour in response to reinforcement, enabling flexibility in the face of changing payoffs [15,51]. Such flexibility also illustrates the benefits of tracking and responding to others’ behaviour in dynamic social environments, a key assumption of the Social Intelligence Hypothesis [52]. However, this need not involve specialized or so-called ‘higher’ cognitive processes [21,53]. We found that the observed effects of experience on behaviour at *experimental arrays* held regardless of whether we considered only co-occupations where adults joined juveniles or also included those where juveniles joined adults. This indicates that in either case adults could learn, from past experience, to associate the presence of juveniles with rewards, consistent with the growing number of studies illustrating that associative learning alone can enable the strategic modification of social behaviour across a range of contexts [21,54,55]. In addition, generalizing among similar individuals could facilitate access to valuable new opportunities and information without needing to remember the outcomes of past interactions with many different individuals, reducing cognitive burdens associated with sampling large numbers of potential social partners [21].

It is also likely that different adults implemented different strategies. The changes in interpretation when removing the individual responsible for the largest number of successful joining events, i.e. strong evidence for generalizing across juveniles when considering displacements but greater evidence for the importance of experience with specific individuals when considering co-occupation—highlights the potential for variation in who is attended to. In practice, it is likely that both remembering past interactions with specific individuals and generalizing across groups of individuals will be used together to guide behaviour, although the former may be a more common daily occurrence than the latter. Differences in how individuals integrate these processes could be underpinned by differences in cognitive abilities or prior experience [56], but we do not have a sample size large enough to investigate these differences robustly. It is also possible that the behaviour of juveniles changed in response to adults (e.g. vacating feeders more when seeing an adult if they had previously been displaced), but the nature of our RFID-based experiment did not enable the collection of such behavioural data. Regardless of the specific strategy implemented by individuals, the key finding that adults learn to modify their social tolerance is clear: results cannot be explained by adults’ pre-existing preference for associating with juveniles, or their propensity to engage with the experiment in general (electronic supplementary material, Initial Propensity).

We show that by adjusting social tolerance adult jackdaws were able to exploit a new resource. More broadly, such flexibility could facilitate the flow of information through networks by permitting close observation of a wide range of innovators. Indeed, the ability to capitalize on opportunities generated by innovators through flexible information use is thought to be a crucial element of human culture [15]. Assessing the degree of flexibility in social information use and social learning by non-human animals will be an important factor in understanding how they respond to changes in the environment and increasing interactions with humans [57]. Our work also provides a rare illustration of the feedback between information use and social structure in natural populations, contributing to our understanding of how sociality and decision-making processes coevolve [58,59].

## Data Availability

All data and code for this work are available as part of the electronic supplementary material. Supplementary material is available online [60].
